# Theoretical Spectroscopic Study of Two Ketones of Atmospheric Interest: Methyl Glyoxal (CH_3_COCHO) and Methyl Vinyl Ketone (CH_3_COCH=CH_2_)

**DOI:** 10.1021/acs.jpca.2c05653

**Published:** 2022-09-30

**Authors:** Insaf Toumi, Samira Dalbouha, Muneerah Mogren Al-Mogren, Ounaies Yazidi, Nejm-Eddine Jaïdane, Miguel Carvajal, María Luisa Senent

**Affiliations:** † Laboratoire de Spectroscopie Atomique Moléculaire et Applications, Faculté des Sciences de Tunis, Université de Tunis El Manar, 2092 Tunis, Tunisia; ‡ Laboratoire de Spectroscopie, Modélisation Moléculaire, Matériaux, Nanomatériaux, Eau et Environnement, LS3MN2E/CERNE2D, Faculté des Sciences Rabat, Université Mohammed V de Rabat, BP 1014 Rabat, Morocco; § Laboratoire de Chimie Organique et de Chimie Physique, Equipe de recherche: Modélisation Moléculaire, Matériaux et Environnement, Département de chimie, Faculté des Sciences d’Agadir, Université Ibn Zohr d’Agadir, BP 8106 Agadir, Morocco; ∥ Chemistry Department, Faculty of Science, King Saud University, P.O. Box 2455, Riyadh 11451, Kingdom of Saudi Arabia; ⊥ Institut Préparatoire aux Etudes d’Ingénieurs el Manar, Université de Tunis El Manar, BP 244, 2092 Tunis, Tunisia; # Departamento de Ciencias Integradas, Centro de Estudios Avanzados en Física, Matemática y Computación, Unidad Asociada GIFMAN, CSIC-UHU, Universidad de Huelva, 21071 Huelva, Spain; ○ Departamento de Química y Física Teóricas, Instituto de Estructura de la Materia, IEM-CSIC, Serrano 121, 28006 Madrid, Spain; ∇ Instituto Universitario Carlos I de Física Teórica y Computacional, Universidad de Granada, 18071 Granada, Spain; @ Unidad Asociada GIFMAN, CSIC-UHU, Universidad de Huelva, 21071 Huelva, Spain

## Abstract

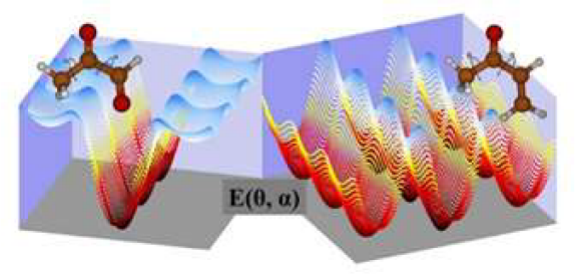

Two ketones of atmospheric interest, methyl glyoxal and methyl vinyl ketone, are studied using explicitly correlated coupled cluster theory and core–valence correlation-consistent basis sets. The work focuses on the far-infrared region. At the employed level of theory, the rotational constants can be determined to within a few megahertz of the experimental data. Both molecules present two conformers, trans/cis and antiperiplanar (A_p_)/synperiplanar (S_p_), respectively. *trans*-Methyl glyoxal and A_p_-methyl vinyl ketone are the preferred structures. *cis*-Methyl glyoxal is a secondary minimum of very low stability, which justifies the unavailability of experimental data in this form. In methyl vinyl ketone, the two conformers are almost isoenergetic, but the interconversion implies a relatively high torsional barrier of 1798 cm^–1^. A very low methyl torsional barrier was estimated for *trans*-methyl glyoxal (*V*_3_ = 273.6 cm^–1^). Barriers of 429.6 and 380.7 cm^–1^ were computed for A_p_- and S_p_-methyl vinyl ketone. Vibrational second-order perturbation theory was applied to determine the rovibrational parameters. The far-infrared region was explored using a variational procedure of reduced dimensionality. For *trans*-methyl glyoxal, the ground vibrational state was estimated to split by 0.067 cm^–1^, and the two low excited energy levels (1 0) and (0 1) were found to lie at 89.588 cm^–1^/88.683 cm^–1^ (A_2_/E) and 124.636 cm^–1^/123.785 cm^–1^ (A_2_/E). For A_p_- and S_p_-methyl vinyl ketone, the ground vibrational state splittings were estimated to be 0.008 and 0.017 cm^–1^, respectively.

## Introduction

Atmospheric processes involving volatile organic compounds (VOCs), such as the oxidation of isoprene (C_5_H_8_) by hydroxyl radicals and ozone, lead to the formation of methyl vinyl ketone (MVK, CH_3_COCHCH_2_, butenone), further oxidation of which produces methyl glyoxal (CH_3_COCHO) and other products.^1,2^ Methyl glyoxal has been recognized as an important precursor of secondary organic aerosols (SOAs) through an atmospheric heterogeneous process.^3^ It has a short lifetime (∼2 h in daytime) and can also be taken up by aqueous aerosols and cloud droplets on account of their high water solubility.^3^

Thus, methyl glyoxal, which is a reduced derivative of pyruvic acid,^1^ represents an atmospherically important dicarbonyl compound produced in the atmosphere from the oxidation of a number of biogenic and anthropogenic VOCs.^3^ When nitrogen oxide abundances are low, acetone is an efficient secondary precursor.^3^ UV photolysis and the reaction with hydroxyl radical are the main gas-phase loss processes for methyl glyoxal^1^ and yield carbon monoxide, acetaldehyde, and formaldehyde.

On the other hand, MVK can originate from the primary emissions produced by fuel evaporation and combustion in urban areas and by biomass burning.^4,5^ For short unsaturated ketones, atmospheric destruction is mostly controlled by the reaction with OH radicals.^6^ Atmospheric oxidation of unsaturated ketones produces a variety of products following the addition of the oxidant to the double bond. Both photolysis and reaction with OH radicals are very effective loss processes for unsaturated dicarbonyls, leading to lifetimes of a few hours. The reaction of MVK with OH has been investigated in atmospheric simulation chambers.^7^

Atmospheric research on pollutants requires previous laboratory studies contemplating structural and spectroscopic properties and chemical processes. Different theoretical and experimental techniques can be employed.^6^ Ab initio calculations can help the interpretation of the observations, providing viewpoints that are especially relevant for features that are difficult to address experimentally. Unfortunately, few previous works have been devoted to methyl glyoxal and MVK. The first electronic transitions of α,β-dicarbonyls, such as glyoxal and its methyl derivatives, have been extensively studied.^8−12^ Franck–Condon analysis of methyl glyoxal shows unambiguously that excitation from the ground state to the lowest triplet state is accompanied by a rotation of the methyl group. A similar change occurs in the first excited singlet state.^8^ Assignments of the electronic transitions or studies of the photodissociation processes must take into account the torsional structure of the methyl derivatives.^12^ Detailed assignments of the vibronic bands in the dispersed fluorescence spectra due to the S_1_(n, π*) ← S_0_ transition were made. The complicated vibrational structures of the fluorescence and phosphorescence excitation spectra of methyl glyoxal and diacetyl were analyzed in terms of the internal rotational modes of the methyl group and the skeletal modes of the glyoxal framework.

The rotational and vibrational spectra of both methyl glyoxal and MVK have been previously measured and assigned.^13−23^ Both molecules show two stable conformers of *C*_*s*_ symmetry that interconvert through large-amplitude motions, although spectroscopic data are unavailable for *cis*-methyl glyoxal. The rotational spectrum of *trans*-methyl glyoxal was first measured and assigned by Dyllick-Brenzinger and Bauder,^13^ who provided the rotational constants, the components of the electric dipole moment (μ_a_ = 0.1597(11), μ_b_ = 0.9620(7)), and the internal rotation barrier (*V*_3_ = 269.1(3) cm^–1^). The methyl torsional fundamental was predicted at 101.6 cm^–1^ from the results of the internal rotation splittings, although a considerably higher value of 122.7 cm^–1^ was determined from the relative intensity measurements. The authors found the interaction between the methyl and skeletal torsions to be the origin of this discrepancy. Recently, Bteich et al.^14^ measured the rotational spectrum in its ground vibrational state in the 4–500 GHz region. The torsion of the central bond was studied by Fateley et al.^15^ Profeta et al.^16^ provided the pressure-broadened quantitative infrared spectrum covering the 520–6500 cm^–1^ range with a resolution of 0.112 cm^–1^. To complete the vibrational assignments, the far-infrared (FIR) spectrum in the 25–600 cm^–1^ region was reported in the same paper.^16^

Previous experimental^17−23^ and theoretical^24−26^ studies of methyl vinyl ketone are available. The IR and Raman spectra have been described.^18−20^ Two stable geometries, the antiperiplanar (A_p_) and synperiplanar (S_p_) conformers, have been identified, although old studies attended to the most stable conformer A_p_. The rotational spectrum of the A_p_ form was first analyzed in 1965 by Foster et al.^17^ The methyl group internal rotation barrier was estimated to be *V*_3_ = 437.19 cm^–1^ (1250 ± 20 cal/mol) and the electric dipole moment to be 3.16 ± 0.05 D.^17^ The torsional splittings and the interaction between the methyl and skeletal torsions were considered in the work of Fantoni et al.^21^ for the antiperiplanar form. The presence of a very stable secondary minimum was reported for the first time in 2011 in the microwave study of Wilcox et al.^22^ Recently, a rotational study of both conformers was performed by Zakharenko et al.^23^

The present study is based on highly correlated ab initio calculations. The goal is to provide structural and spectroscopic theoretical data with an emphasis on the FIR region following the same procedure employed for the interconnecting carbonyl species pyruvic acid and acetone.^27−29^ We study both molecules in the same paper because they share properties. Both species can be considered as acetone derivatives in which one acetone methyl group has been substituted by another functional group, −CHO or −CH=CH_2_. Both species present two interacting torsional modes. The work represents a step of a general project dealing with atmospheric ketones.

We seek to underline aspects that are not fully understood or are the object of discussion. In methyl glyoxal, the cis structure is almost unidentified, and discrepancies due to the strong coupling between the two torsional modes are present in the previous assignments of the spectra of the trans structure. For both conformers, special attention is given to the FIR region. In MVK, the coupling between large-amplitude motions hinders the assignments. Both species show very low methyl torsional barriers (*V*_3_ < 400 cm^–1^), whose effects make interpretation difficult.

The FIR spectra of the two species are simulated using a variational procedure of reduced dimensionality implemented in an original code that uses data from ab initio calculations as input. This procedure allows us to provide information concerning the large-amplitude motions and the interactions, barriers, and torsional parameters and to map the low-lying vibrational states and their splittings. This information can be useful for the interpretation of measurements in the FIR region and also can help in the assignments of rotational and rovibrational spectra.

## Theoretical Methods

The geometries of the minimum-energy structures and the ab initio potential energy surfaces of reduced dimensionality were computed using explicitly correlated coupled cluster theory with single and double substitutions augmented by a perturbative treatment of triple excitations (CCSD(T)-F12b)^30,31^ as implemented in Molpro^32^ using default options. All of the core and valence electrons were correlated in the post-SCF process. The core–valence correlation-consistent basis set cc-pCVTZ-F12 developed for the explicitly correlated methods was employed as the basis set in connection to the additional basis sets optimized for use in the resolution of the identity.^33^

In all of the work, different levels of theory were selected by taking into consideration the required precision and the available computational tools. Second-order Møller-Plesset perturbation theory (MP2)^34^ as implemented in Gaussian 16, revision C.01^35^ was employed in connection with the aug-cc-pVTZ (AVTZ) basis set^36^ to obtain anharmonic spectroscopic properties and the vibrational corrections for the rotational parameters and potential energy surfaces. The full-dimensional anharmonic force field was determined at all of the minima.

Vibrational second-order perturbation theory (VPT2) as implemented in Gaussian 16, revision C.01^37^ was employed to obtain spectroscopic properties for the low- and medium-amplitude vibrational motions. The two torsional modes were studied variationally using the code ENEDIM^38−40^ following a procedure that allows mapping of the low-lying vibrational energy levels and the splittings.

Throughout the work, to save computational time, different levels of ab initio calculation were employed and combined. First-order parameters (structures, equilibrium rotational constants) were computed using CCSD(T)-F12 theory, whereas the vibrational corrections were determined at a lower level of theory such as MP2. Then the relevant contributions to the observable parameters were obtained and found to be very accurate. Further corrections are less dependent on the correlation energy.

## Results and Discussion

### Structures of the Conformers

**Figure 1:**
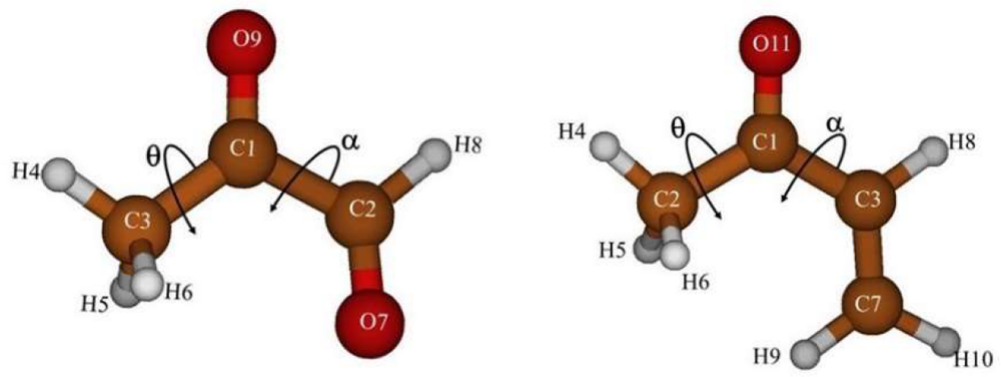
Atom distributions in *trans*-methyl glyoxal and Ap-MVK. The internal rotation coordinates are θ and α.

Both methyl glyoxal and MVK show two conformers, trans/cis and A_p_/S_p_, respectively. *trans*-Methyl glyoxal and A_p_-MVK are the preferred structures. In *trans*-methyl glyoxal, “trans” refers to the relative positions of the two oxygen atoms with respect to the C1–C2 bond. In A_p_-MVK, “antiperiplanar” refers to the relative positions of the two double bonds. In Table 1, the corresponding structural parameters computed at the CCSD(T)-F12/CVTZ-F12 level of theory are collected. For the computation, all of the core and valence electrons were correlated in the post-SCF process. For an easy understanding of Table 1, Figure 1 shows the atom distributions in the most stable geometries.

**Table 1:** CCSD(T)-F12/cc-pCVTZ-F12 Relative Energies (Δ*E* and Δ*E*^ZPVE^, in cm^–1^), Internal Rotation Barriers (*V*_3_ and *V*^α^, in cm^–1^), Rotational Constants (in MHz), MP2/AVTZ Dipole Moments (in D) and Equilibrium Structural Parameters (Distances in Å and Angles in Degrees) for Methyl Glyoxal and Methyl Vinyl Ketone

	CH_3_COCHO		CH_3_COCH=CH_2_	
parameter	trans	cis	A_p_	S_p_
Δ*E*	0.0^a^	1835	0.0^b^	196
Δ*E*^ZPVE^	0.0	1747	0.0	158
θ	0.0	0.0	0.0	0.0
α	180.0	0.0	0.0	180.0
*V*^α^	1980.1		1798.3	
*V*_3_	273.6	361.9	429.6	380.7
*A*_e_	9172.48	10470.07	9015.97	10301.29
*B*_e_	4470.82	4061.31	4316.10	4025.09
*C*_e_	3061.77	2979.74	2972.38	2946.60
μ_a_	0.1703	2.8367	3.1006	0.6066
μ_b_	0.9727	4.2102	2.2772	3.1325
μ	0.9875	5.0767	3.8469	3.1907

CH_3_COCHO (*C*_*s*_)					
	trans	cis		trans	cis
C2–C1	1.5254	1.5416	H5–C3–C1	109.6	109.9
C3–C1	1.4957	1.5015	H6–C3–C1	109.6	109.9
H4–C3	1.0848	1.0847	O7–C2–C1	122.7	122.3
H5–C3	1.0848	1.0913	H8–C2–C1	113.9	115.9
H6–C3	1.0848	1.0913	O9–C1–C2	117.6	119.9
O7–C2	1.2048	1.2003	H4–C3–C1–C2	180.0	180.0
H8–C2	1.1018	1.1052	H5–C3–C1–H4	121.7	121.3
O9–C1	1.2102	1.2042	H6–C3–C1–H4	–121.7	–121.3
C3–C1–C2	117.3	115.7	O7–C2–C1–C3	0.0	180.0
H4–C3–C1	109.7	110.1	H8–C2–C1–C3	180.0	0.0

CH_3_COCH=CH_2_ (*C*_*s*_)					
	A_p_	S_p_		A_p_	S_p_
C2–C1	1.5080	1.5052	H6–C2–C1	110.5	109.7
C3–C1	1.4833	1.4924	C7–C3–C1	124.2	121.0
H4–C2	1.0851	1.0851	H8–C3–C1	114.4	117.7
H5–C2	1.0898	1.0909	H9–C7–C3	121.1	121.6
H6–C2	1.0898	1.0909	H10–C7–C3	121.7	119.7
C7–C3	1.3348	1.4924	O11–C1–C3	119.5	121.7
H8–C3	1.0823	1.0828	H4–C2–C1–C3	180.0	180.0
H9–C7	1.0803	1.0800	H5–C2–C1–H4	120.5	121.3
H10–C7	1.0810	1.0816	H6–C2–C1–H4	–120.5	–121.3
O11–C1	1.2157	1.2133	C7–C3–C1–C2	0.0	180.0
C3–C1–C2	119.1	116.1	H9–C7–C3–C1	180.0	180.0
H4–C2–C1	108.8	110.1	H10–C7–C3–C1	0.0	0.0
H5–C2–C1	110.5	109.7			

a *E*_a_ = −267.134470 au.

b *E*_a_ = −231.225040 au.

**Figure 2:**
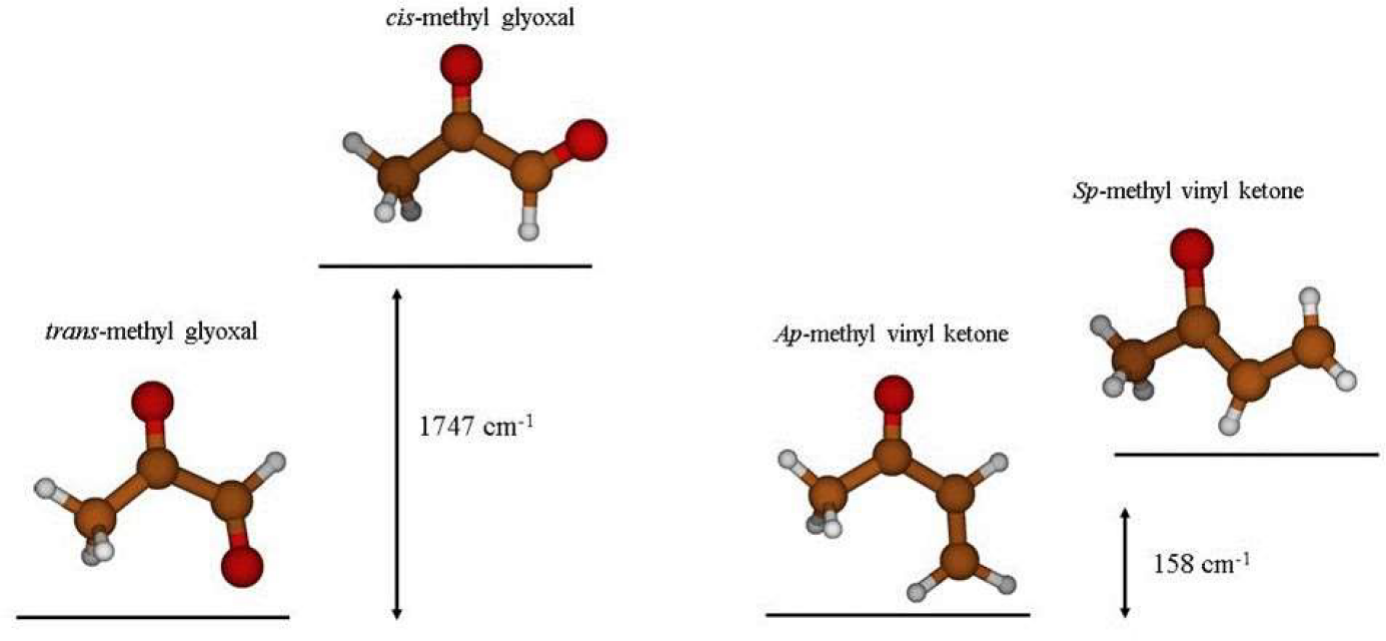
Relative stabilities of the conformers of methyl glyoxal and MVK.

**Figure 3:**
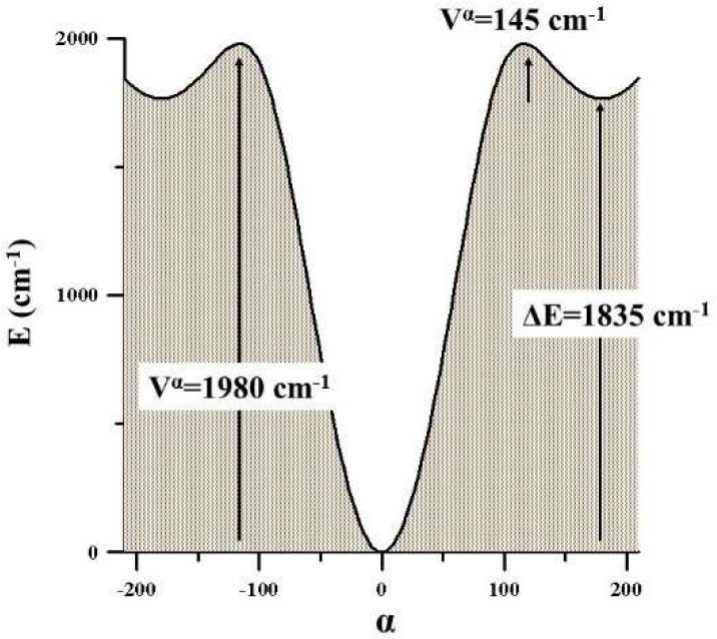
Energy profile for the trans → cis transformation of methyl glyoxal.

**Figure 4:**
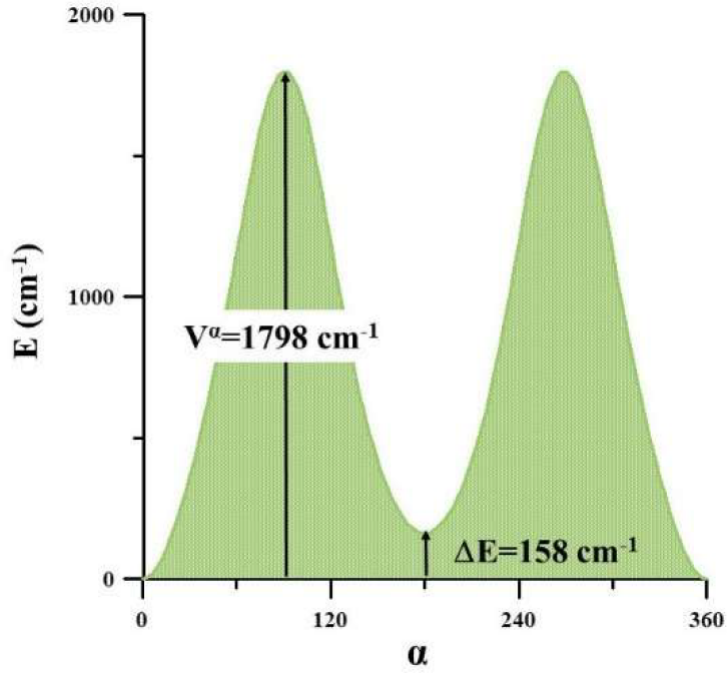
Energy profile for the A_p_ → S_p_ transformation of methyl vinyl ketone.

Table 1 shows the CCSD(T)-F12/CVTZ-F12 equilibrium rotational constants and the MP2/AVTZ components of the electric dipole moment referred to the principal axes system, the relative energies of the conformers, and the two torsional barriers, *V*_3_ (methyl group torsion) and *V*^α^ (central bond torsion). The vibrationally corrected relative energies of the conformers are shown in Figure 2. The energy profiles accompanying the conformer conversions are represented in Figures 3 and 4.

**Figure 5:**
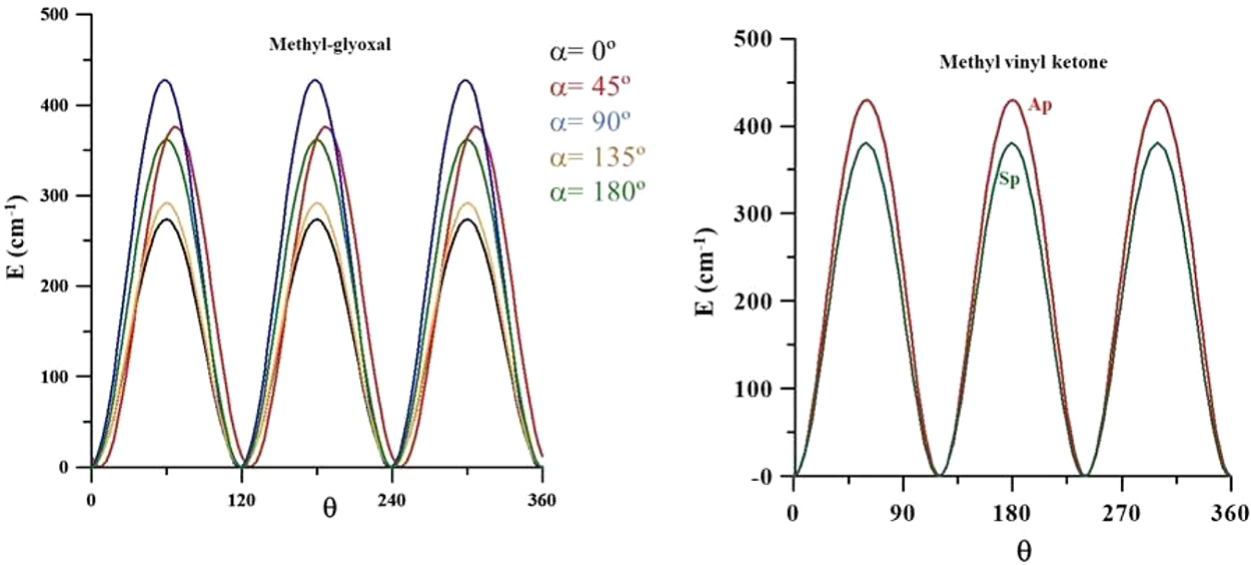
Methyl torsional barriers of methyl glyoxal and methyl vinyl ketone.

For methyl glyoxal, the relative energy between the conformers was computed to be Δ*E* = 1835 cm^–1^, and the relative energy including zero-point vibrational energy was Δ*E*^ZPVE^ = 1747 cm^–1^. The energy profiles shown in Figures 3, 4, and 5 are one-dimensional cuts of the potential energy surfaces described in the next sections. The barrier for the trans → cis process was estimated to be 1980 cm^–1^, assuring the prominent feasibility of the trans form. The inverse cis → trans process, which is restricted by a very low barrier of ∼150 cm^–1^, can occur at very low temperatures. This draws a secondary cis minimum of very low stability and justifies the unavailability of experimental data for the cis form.

On the other hand, in MVK the A_p_ → S_p_ process shown in Figure 4 is restricted by a barrier of 1798 cm^–1^. The A_p_ and S_p_ conformers are almost isoenergetic, but their interconversion is hampered by the relatively high torsional barrier.

The methyl torsional barriers of *trans*- and *cis*-methyl glyoxal were computed to be 273.6 and 361.9 cm^–1^ at the CCSD(T)-F12/CVTZ-F12 level of theory. For the trans form, the barrier is in very good agreement with the experimental value of 271.718(24) cm^–1^.^14^ In A_p_- and S_p_-MVK, these parameters were estimated to be 429.6 and 380.7 cm^–1^, respectively, which can be compared with the experimental values of 443.236(78) and 385.28(30) cm^–1^, respectively.^23^ Contrary to what is usual, there is better agreement between the calculated and experimental values for the secondary minimum than for the preferred one. It must be considered that the two structures are almost isoenergetic.^41^Figure 5 shows the energy variation with the internal rotation of the methyl groups computed for different α values. Differences between the profiles denote the potential interactions between the two torsional modes of each molecule.

In a previous work devoted to acetone,^28^ the computed methyl torsional barrier of acetone (*V*_3_ = 246 cm^–1^)^28^ was compared with those of methyl formate (*V*_3_ = 368 cm^–1^)^42^ and dimethyl ether (*V*_3_ = 951 cm^–1^).^43^ The theoretical work,^28^ which was performed using highly correlated ab initio methods, highlighted the effects derived from the very low barrier of acetone, which made the computation of the low vibrational energy levels and the force field using numerical derivatives challenging. Effects that made the experimental spectrum assignments difficult were also observed (see ref (28) and references therein). Computations of the rovibrational parameters were less problematic for methyl formate^42^ and dimethyl ether,^43^ for which the barriers are higher than in acetone. Thus, *trans*-methyl glyoxal can be expected to behave like acetone. In MVK, the barriers are like that of methyl formate.

### Rovibrational Parameters

The ground vibrational state rotational constants *B*_0_ shown in Table 2 were computed using the following equation and the CCSD(T)-F12/CVTZ-F12 equilibrium rotational constants:
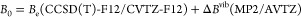
1where Δ*B*^vib^ is the vibrational contribution derived from the VPT2 α_r_^*i*^ vibration–rotation interaction parameters determined from the MP2/AVTZ cubic force field. In Table 2, the computed results are compared with experimental results. All of these experimental parameters are referred to the principal axis system, although different authors employed different definitions of the reference axis and effective Hamiltonians (principal axis method (PAM), rho axis method (RAM), or internal axis method (IAM)).

**Table 2:** Ground Vibrational State Rotational Constants (in MHz) Referred to the Principal Axis System^a^

Methyl Glyoxal				
	*trans*			*cis*
constant	calcd	exptl^b^	exptl^c^	calcd
*A*_0_	9103.21	9102.4332(31)	9108.41(15)	10391.75
*B*_0_	4438.75	4439.8832(27)	4445.48(15)	4032.95
*C*_0_	3039.10	3038.9404(22)	3036.778(60)	2963.05

A_p_-Methyl Vinyl Ketone					
constant	calcd	exptl^d^	exptl^e^	exptl^f^	exptl^g^
*A*_0_	8940.41	8941.45(6)	8941.58(1)	8941.547(1)	8924.9
*B*_0_	4275.17	4274.48(2)	4274.494(5)	4274.3593(9)	4276.8
*C*_0_	2945.18	2945.32(2)	2945.276(4)	2945.2903(9)	2942.9

S_p_-Methyl Vinyl Ketone			
constant	calcd	exptl^f^	exptl^g^
*A*_0_	10236.60	10238.610(1)	10220.5
*B*_0_	3993.15	3991.6814(6)	3996.8
*C*_0_	2926.75	2925.4885(1)	2926.2

a All of the experimental parameters have been transformed to be referred to the principal axis following the methods of refs (23) and (44) from the original axis systems, as indicated.

b PAM, ref (13).

c RAM, ref (14).

d PAM, ref (17).

e PAM, ref (21).

f IAM, ref (22).

g RAM, ref (23).

It is worth noting the very good agreement between the computed and experimental data for the three rotational constants. In previous studies, we determined the parameters discriminating valence and core electrons. Correlation effects due to the valence electrons were evaluated at the CCSD(T)-F12 level of theory, while CCSD(T) theory was used to describe the core correlation effects.^27−29,45,46^ Generally, the approximation led to an error in one of the constants larger than for the other two parameters. In the present case, both correlation effects were treated together using CCSD(T)-F12 and a suitable basis set. In the absence of high-resolution IR data, the microwave parameters are the only experimental values to be compared with calculations. This at least qualitatively gives an estimation of the accuracy one should expect for the FIR predictions described in the next sections.

The computed parameters are closer to the available experimental ones derived with the PAM and IAM methods (|*B*^calcd^ – *B*^exptl^| < 2 MHz) than those obtained using RAM. This last fitting procedure is more suitable for species showing low internal rotation barriers. The differences are similar for methyl glyoxal, showing a lower barrier than for MVK. They can be related to the number of fitting parameters, to the MP2/AVTZ force field accuracy, or to the theoretical procedure applied for the computation of the α_r_^*i*^ vibration–rotation interaction parameters developed for semirigid systems. In addition to a different model Hamiltonian, the data from refs (14) and (23) include many more and much higher energy levels obtained from microwave spectra. The constants are then expected to be more accurate but are also effective due to the RAM Hamiltonian.

Table 3 collects the quartic centrifugal distortion constants. For *trans*-methyl glyoxal, the computed values are compared with those of ref (13) obtained using the Watson asymmetrically reduced Hamiltonian.^47^ Some discrepancies with the experimental data are relevant for Δ_*J*_ and Δ_*K*_, we omit the sextic constants. The quartic centrifugal distortion constants are provided to complete the theoretical information on this research.

**Table 3:** MP2/AVTZ Quartic Centrifugal Distortion Constants^a^ (in kHz) Computed Using the MP2/AVTZ Cubic Force Field

	CH_3_COCHO			CH_3_COCH=CH_2_	
	trans		cis	A_p_	S_p_
constant	calcd	exptl^13^	calcd	calcd	calcd
Δ_*J*_	1.0422	1.327(39)	0.8214	0.7953	0.7329
Δ_*K*_	–2.2290	–1.41(70)	8.4899	0.8428	7.7405
Δ_*JK*_	7.905	7.18(22)	4.8110	4.3781	2.9267
δ_*J*_	0.3164	0.464(12)	0.2274	0.2434	0.2057
δ_*K*_	4.5508	5.68(18)	3.0157	2.9206	2.1177

a Asymmetrically reduced Hamiltonian; IIIr representation.

### Vibrational Fundamentals

The vibrational energies for all of the vibrational modes were computed using the following equation:
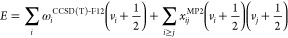
2where the ω_*i*_ are the harmonic fundamentals, *v*_*i*_ and *v*_*j*_ are vibrational quanta, and *x*_*ij*_ are the anharmonic constants. The “harmonic contribution” was obtained using CCSD(T)-F12/AVTZ-F12, whereas the anharmonic constants were derived using VPT2 and the MP2/AVTZ force field. The anharmonic fundamentals are shown in Table 4.

**Table 4:** Anharmonic Fundamental Frequencies (in cm^–1^)^a^ Calculated in This Work and Measured in Previous Experiments in the Gas Phase

CH_3_COCHO (*C*_*s*_)				
		*trans*		*cis*
mode	assignment	calcd	exptl^16^	calcd
A′				
1	CH_3_ st	**3029**	3026.27	3028
2	CH_3_ st	2939	2950.07	2228
3	CH st	2829	2828.01	**2872**
4	CO st	**1738**	1733.27	**1774**
5	CO st	1733	1729.41	**1755**
6	CH_3_ b	1420	1422.92	1428
7	CH_3_ b	1364	1367.38	1377
8	COH b	**1323**	1265.57	1360
9	CC st	**1228**	1228.81	**1159**
10	CH_3_ b	**1002**	1005.65	**953**
11	CC st	776	781.23	**810**
12	OCC b	**567**	535.21; 591.22	634
13	CCO b	475	477.63	398
14	CCC b	268	257.76	**272**
A″				
15	CH_3_ st	2977	2977.85	2965
16	CH_3_ b	**1416**	1425.22	**1433**
17	CCC b	1050	1052.04	1045
18	CO w	**881**	887.12	**869**
19	CCO b	454	480.04	**457**
20	CH_3_ tor	126	121.09^16^	121
21	CC tor	96	103;^16^ 105 ± 2;^15^ 89^11^	41

CH_3_COCH=CH_2_							
		A_p_			S_p_		
			exptl^19^			exptl^19^	
mode	assignment	calcd	IR	Raman	calcd	IR	Raman
A′							
1	CH_2_ st	3105	3105	3105	3111		
2	CH_2_ st	**3058**	3036	3036	**3061**		
3	CH st	3021	3019	3017	3023		
4	CH_3_ st	**2993**		2996	**3040**		
5	CH_3_ st	2929	2936	2937	2921		
6	C=O st	**1706**	1705	1705	1730	∼1729	1721
7	C=C st	1624	1620	1623	1623		
8	CH_3_ b	**1449**		1426	**1427**		
9	CH_2_ b	**1420**	1400	1404	1395		
10	CH_3_ b	1359	1366		1352	1355	
11	CH b	**1272**		1283	1295		1298
12	CCC st	**1244**	1249	∼1257	**1175**	1218	
13	CH_2_ w	1052	1062	1062	1064	1180	
14	CH_3_ b	935	1026		950		
15	CCC st	753	758	758	771	772	772
16	H_2_CCC def	**533**	530		598	609	
17	ske def	**478**	492		410		
18	ske def	**311**	(old 413);^19^ 292^b^		**273**	(old 422);^19^ 272^b^	
A″							
19	CH_3_ st	2978	2971	∼2955	2967		
20	CH_3_ b	1434			**1444**		
21	CH_3_ b	**1016**	1002		1014		
22	CH_3_ b	995	987		998		
23	CH_2_ b	954	950	948	973	968	
24	CH b	679	691		**658**	662	
25	OCC def	**416**	(old 292);^19^ 413^b^	291	**439**	(old 272);^19^ 422^b^	273
26	CH_3_ tor	124	125		123	121	
27	CC tor	98	116^b^		69	87	

a Mode abbreviations: st = stretching; b = bending; w = wagging; tor = torsion; def = deformation; ske def= skeletal deformation. Fermi displacements have been considered. The bands that are strongly affected by the interactions are emphasized in boldface type.

b New assignments proposed in this work.

The calculated values are compared with available experimental data from refs (11), (15), and (16) in the case of methyl glyoxal and from ref (19) in the case of methyl vinyl ketone. In general, there is an expectable agreement between computations and observations for the medium and high frequencies. An exception ensues for the modes ν_18_ and ν_25_ that were assigned in ref (19) to the bands observed at 413 and 292 cm^–1^ for A_p_-MVK and 422 and 272 cm^–1^ for S_p_-MVK, respectively. Our computed values are ν_18_ = 311 and 273 cm^–1^ and ν_25_ = 416 and 439 cm^–1^. Thus, on the basis of the calculations, we propose the new assignment of these two bands altering the two designated values.

Fermi displacements were predicted using VPT2 as implemented in Gaussian 16^35^ and the cubic force field. Those displacements were considered to obtain the values shown in Table 4. The bands that are strongly affected by the interactions are emphasized in boldface type.

Previous studies of *trans*-methyl glyoxal evidence disagreements for the assignments of the ν_20_ and ν_21_ torsional modes because for both normal modes the methyl and C–C CH_3_ torsions present strong interactions.^13^ Therefore, separability of the two torsions is not feasible. This was highlighted in ref (14), attending to the displacement **L** matrices. Then the lowest-frequency mode, assigned in ref (11) to the methyl torsional mode, is attributed in ref (14) to the C–C torsion. The present results and the analysis of the internal coordinate contributions to the normal modes, as well as the wave functions derived from the variational procedure, confirm the calculations of Bteich et al.,^14^ which are in good agreement with the assignments of Profeta et al.^16^ To obtain a better understanding, we determined the band positions for three different isotopic varieties using the variational procedure described below. In Table 5, the resulting frequencies are compared with harmonic frequencies computed using the MP2/AVTZ force field. Those results allow us to assign ν_20_ and ν_21_ to the methyl and C–C torsional modes, respectively.

**Table 5:** CCSD(T)-F12/CVTZ-F12 Torsional Band Center Positions (ν, in cm^–1^) Computed Variationally for Three Different Isotopologues and MP2/AVTZ Harmonic Frequencies (ω, in cm^–1^)

	CH_3_COCHO		CD_3_COCHO		CH_3_COCD^18^O	
mode	ω	ν (variational)	ω	ν (variational)	ω	ν (variational)
ν_20_	131	124.6	123	117.6	126	116.8
ν_21_	99	89.6	77	69	95	85.0

### Far-Infrared Region

**Figure 6:**
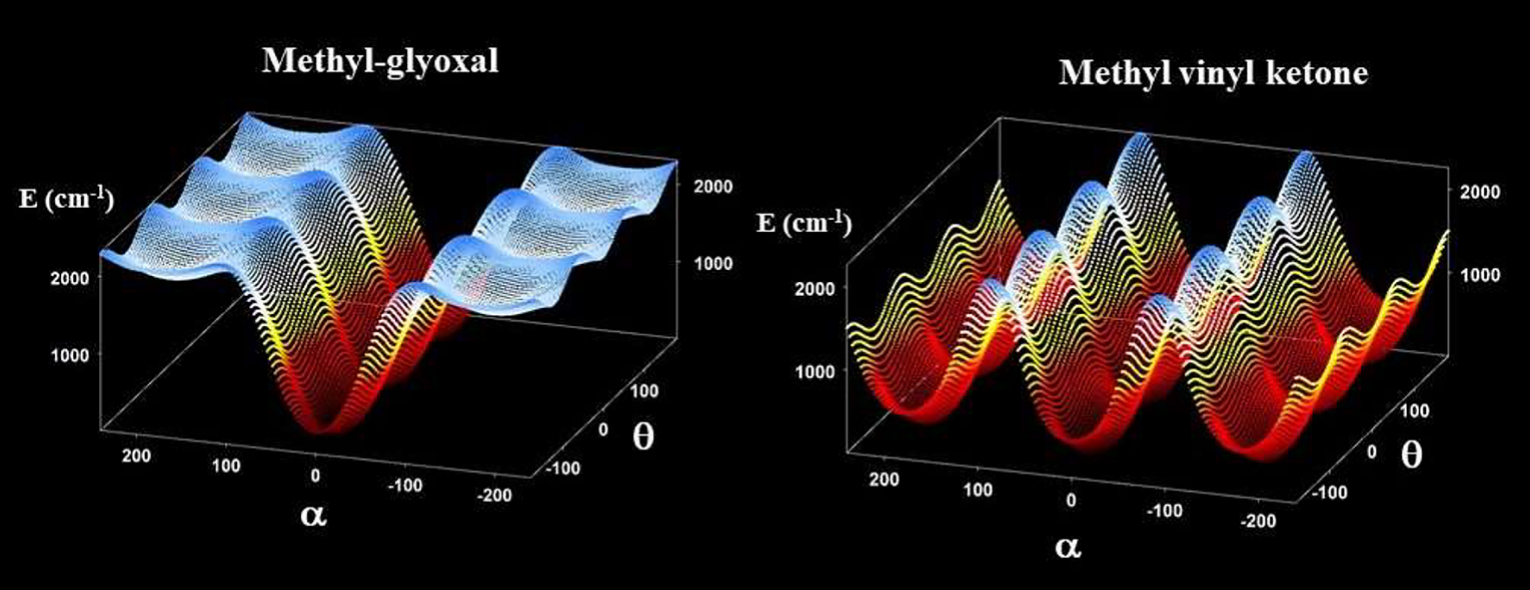
Two-dimensional potential energy surfaces of the methyl-glyoxal and methyl vinyl ketone.

The low-lying vibrational energy levels corresponding to the two torsional modes, the methyl torsion θ and the C–C torsion α, were obtained by solving the following Hamiltonian variationally:^38−40^
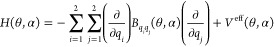
3This Hamiltonian was defined by assuming the separability of the two torsional modes from the remaining vibrational modes on the basis of vibrational energies and the predicted resonances derived from VPT2. In eq 3, *B*_*q_i_q_j_*_ and *V*^eff^ are the kinetic energy parameters and the effective potential, which is defined as the sum of three contributions:
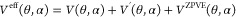
4where *V*(θ, α) is the ab initio two-dimensional potential energy surface, *V*′(θ, α) is the Podolsky pseudopotential, and *V*^ZPVE^(θ, α) is the zero-point vibrational energy correction. The θ and α coordinates are defined as linear combinations of curvilinear internal coordinates. For methyl glyoxal, θ and α are defined as follows:
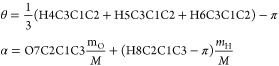
5where *m*_H_ and *m*_O_ are the atomic masses of H and O atoms, respectively, and *M* = *m*_O_ + *m*_H_. For methyl vinyl ketone, θ and α are defined as follows:
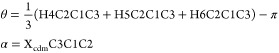
6where X_cdm_ is the center of mass of the vinyl group CH=CH_2_. The two ab initio potential energy surfaces were computed using the CCSD(T)-F12/CVTZ-F12 total electronic energies of 26 geometries defined for different values of θ (0°, 90°, 180°, −90°) and α (0°, 30°, 60°, 90°, 120°, 150°, 180°). In all of these geometries, 3*N*_a_ – 8 internal coordinates (where *N*_a_ is the number of atoms) were allowed to relax at the MP2/AVTZ level of theory. The energies were fitted to the following double Fourier series:
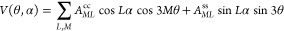
7This function transforms as the totally symmetric representation of the *G*_6_ molecular symmetry group.^29,48^ Formally identical expressions were employed for *V*′, *V*^ZPVE^, *V*^eff^, and the kinetic terms. Details concerning the computation of the Hamiltonian parameters from ab initio energies and geometries can be found in refs (39) and (40). *V*^ZPVE^ was computed using the MP2/AVTZ harmonic fundamentals calculated at all 26 geometries. Figure 6 presents the two effective surfaces.

Thus, for methyl glyoxal, the following expression for *V*^eff^ was obtained:
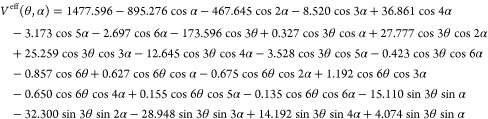
8Equations formally identical to eq 8 were employed for the three kinetic parameters. For methyl glyoxal, the coefficients *A*_00_^cc^ were computed to be *B*_θ_ = 5.5841 cm^–1^, *B*_α_ = 2.2379 cm^–1^, and *B*_θα_ = −0.1861 cm^–1^.

For methyl vinyl ketone, the following expression for *V*^eff^ was obtained:
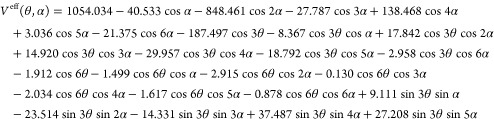
9For methyl vinyl ketone, the coefficients *A*_00_^cc^ were computed to be *B*_θ_ = 5.5885 cm^–1^, *B*_α_ = 1.7971 cm^–1^, and *B*_θα_ = −0.1898 cm^–1^.

The energy levels shown in Tables 6 and 7 were determined by diagonalizing the Hamiltonian matrices, which factorize into blocks due to the symmetry conditions. The levels are classified using the symmetry representations of the *G*_6_ molecular symmetry group, and the two quanta represent excitations of the C–C and methyl torsional modes. They are compared with available experimental data and with the results obtained using VPT2.

For *trans*-methyl glyoxal, the splitting of the ground vibrational state was estimated to be 0.067 cm^–1^. The two lowest excited energy levels, (1 0) and (0 1), were computed to be 89.588 cm^–1^ (A_2_) and 88.683 cm^–1^ (E) and 124.636 cm^–1^(A_2_) and 123.785 cm^–1^ (E). The low-lying energies of *cis*-methyl glyoxal are also provided in Table 6, but as has been already highlighted, the viability of this conformer is very low.

However, in MVK the two conformers show very similar stabilities. As they are separated by a relatively high barrier, the energies can be allocated as if they were two different molecules. In MVK, both modes υ_27_ and υ_26_ can be assigned as the torsional modes without any confusion. For A_p_-MVK, the splitting of the ground vibrational state was estimated to be 0.008 cm^–1^. The two low excited energy levels (1 0) and (0 1) were computed to be 105.344 cm^–1^ (A_2_) and 105.354 cm^–1^ (E) and 137.217 cm^–1^ (A_2_) and 136.869 cm^–1^ (E). For S_p_-MVK, the splitting of the ground vibrational state was estimated to be 0.017 cm^–1^, and the two lowest excited energy levels (1 0) and (0 1) were computed to be 81.940 cm^–1^ (A_2_) and 81.954 cm^–1^ (E) and 127.732 cm^–1^ (A_2_) and 127.130 cm^–1^ (E).

The two conformers A_p_-MVK and S_p_-MVK show very similar *V*_3_ barriers, which were computed to be 429.6 and 380.7 cm^–1^, respectively. On the basis of the barriers, similar splittings of the ground vibrational state will be expected. However, the ZPVEs are very different (128.389 cm^–1^ for A_p_ and 271.375 cm^–1^ for S_p_), and when they are considered, it is easy to understand that the S_p_ splitting is double the A_p_ splitting.

## Conclusions

In this study, the far-infrared spectra of methyl glyoxal and methyl vinyl ketone have been simulated using a variational procedure of reduced dimensionality starting from geometries and potential energy surfaces computed using highly correlated ab initio calculations. The procedure allows us to derive theoretical information concerning the large-amplitude motions and their interactions. Torsional barriers and parameters are provided. The low-lying vibrational states and their splittings have been mapped. We believe that this information can be useful for the interpretation of further measurements in the far-infrared region and also can help with assignments of rotational and rovibrational spectra.

The energy difference between the *trans*- and *cis*-methyl glyoxal conformers has been estimated to be 1747 cm^–1^. The profile of the trans → cis process draws a secondary cis minimum of very low stability and justifies the unavailability of experimental data for the cis form. The A_p_ and S_p_ conformers of methyl vinyl ketone are almost isoenergetic, but their interconversion is hampered by a relatively high torsional barrier of 1798 cm^–1^. The methyl torsional barriers of *trans*-methyl glyoxal, A_p_-MVK, and S_p_-MVK have been computed to be 273.6, 429, and 380.7 cm^–1^, respectively.

Previous studies of methyl glyoxal showed disagreements for the assignments of the ν_20_ and ν_21_ normal modes because they cannot be understood in terms of two different local modes, one describing the CH_3_ internal rotation and the other describing the C–C central bond torsion. The two internal rotations interact strongly and contribute to the two normal modes. The comparison of the computed far-infrared spectra of three different isotopic varieties helped to correlate ν_20_ to the methyl torsion and ν_21_ to the C–C torsion.

The low-lying torsional energy levels of methyl glyoxal and methyl vinyl ketone were computed variationally up to 450 cm^–1^. The energies were assigned to the two conformers of methyl glyoxal and to the two conformers of MVK. The first excited levels of *cis*-methyl glyoxal have been computed, although the lifetime of this structure is very short. For *trans*-methyl glyoxal, the splitting of the ground vibrational state has been estimated to be 0.067 cm^–1^. The two lowest excited energy levels (1 0) and (0 1) were computed to be 89.588/88.683 cm^–1^ (A_2_/E) and 124.636/123.785 cm^–1^ (A_2_/E). For MVK the splittings of the ground vibrational state have been estimated to be 0.008 cm^–1^ (A_p_) and 0.017 cm^–1^ (S_p_). The two lowest excited energy levels (1 0) and (0 1) of A_p_-MVK were computed to be 105.344/105.354 cm^–1^ (A_2_/E) and 137.217/136.869 cm^–1^ (A_2_/E). For S_p_-MVK, the corresponding energies are 81.940/81.954 cm^–1^ (A_2_/E) and 127.732/127.130 cm^–1^ (A_2_/E).

**Table 6:** Low-Lying Vibrational Energy Levels of Methyl Glyoxal (in cm^–1^)

		*trans-*methyl glyoxal			*cis*-methyl glyoxal	
υ_21_ υ_20_	symmetry	variational	VPT2	exptl	variational	VPT2
0 0	A_1_	0.000			0.000	
	E	0.067			0.0025	
1 0	A_2_	89.588	96	89^11^	48.111	41
	E	88.683		103^16^	48.134	
0 1	A_2_	124.636	126	105 ± 2^15^	123.257	121
	E	123.785		121.09^16^		
2 0	A_1_	167.682	183	167^11^	94.668	80
	E	174.832		200^11^		
1 1	A_1_	198.835	213		164.690	153
	E	202.450				
0 2	A_1_	242.736	229		172.366	217
	E	245.715				
3 0	A_2_	261.801	260			117
	E	235.353				
υ_14_	CCC b		268	257.76^16^		262
2 1	A_2_	291.883	291			184
	E	276.655				
4 0	A_1_	296.141	327			152
	E	339.409				
1 2	A_2_	310.899	326			252
	E	310.999				
υ_21_ υ_14_			358			
0 3	A_2_	356.830	364			
	E	360.716				
3 1	A_1_	360.897	360			
	E	363.266				
υ_20_ υ_14_			375			
2 2	A_1_	397.857	396			
	E	378.421				
ZPVE		110.66			1857.52	

**Table 7:** Low-Lying Vibrational Energy Levels of Methyl Vinyl Ketone (in cm^–1^)

		A_p_-methyl vinyl ketone			S_p_- methyl vinyl ketone		
υ_27_ υ_26_	symmetry	variational	VPT2	exptl^19^	variational	VPT2	exptl^19^
0 0	A_1_	0.000	0		0.000	0	
	E	0.008			0.017		
1 0	A_2_	105.344	98	116	81.940	69	87
	E	105.354			81.954		
0 1	A_2_	137.217	124	125	127.732	123	121
	E	136.869			127.130		
2 0	A_1_	207.028	190		162.893	135	
	E	207.052			162.905		
1 1	A_1_	237.694	217		207.448	190	
	E	237.270			207.079		
0 2	A_1_	248.114	206		229.754	225	
	E	253.017			236.405		
3 0	A_2_	304.548	274		243.425	203	
	E	304.599					
υ_18_	ske def		311	292^a^		272	272^a^
2 1	A_2_	344.367	303		286.179	255	
	E	349.558			285.965		
1 2	A_2_	355.026	322		308.934	298	
	E	337.469			308.977		
0 3	A_2_	360.806	332		342.506	331	
	E	327.644			397.294		
4 0	A_1_	383.467	352		323.923	268	
	E	436.458			323.938		
υ_27_ υ_18_			383			331	
0 4	A_1_	397.545	416		421.587	416	
	E	397.415			417.881		
υ_26_ υ_18_			412			390	
2υ_27_ υ_18_						397	
υ_25_	OCC def		416	413^a^		439	422^a^
3 1	A_1_	428.394	382		364.709	319	
	E	416.985			364.354		
2 2	A_1_	443.195	402		387.659	360	
	E	433.235			380.719		
1 3	A_1_	455.580	414		360.184	393	
	E	448.246			317.491		
ZPVE		128.389			271.375		

a New assignments proposed in this work.
